# Macrophage polarization and acceleration of atherosclerotic plaques in a swine model

**DOI:** 10.1371/journal.pone.0193005

**Published:** 2018-03-21

**Authors:** Seul-Gee Lee, Jaewon Oh, Sung-Kyung Bong, Jung-Sun Kim, Seil Park, Sehoon Kim, Sungha Park, Sang-Hak Lee, Yangsoo Jang

**Affiliations:** 1 Graduate Program in Science for Aging, Yonsei University, Seoul, Korea; 2 Severance Cardiovascular Hospital, Cardiovascular Research Institute, Yonsei University College of Medicine, Seoul, Korea; 3 Cardiovascular Product Evaluation Center, Yonsei University College of Medicine, Seoul, Korea; 4 Severance Biomedical Science Institute, Yonsei University College of Medicine, Seoul, Korea; 5 Department of Pathology, Yonsei University College of Medicine, Seoul, Korea; Nagoya University, JAPAN

## Abstract

**Aims:**

Atherosclerosis is a well-known cause of cardiovascular disease and is associated with a variety of inflammatory reactions. However, an adequate large-animal model of advanced plaques to investigate the pathophysiology of atherosclerosis is lacking. Therefore, we developed and assessed a swine model of advanced atherosclerotic plaques with macrophage polarization.

**Methods:**

Mini-pigs were fed a 2% high-cholesterol diet for 7 weeks followed by withdrawal periods of 4 weeks. Endothelial denudation was performed using a balloon catheter on 32 coronary and femoral arteries of 8 mini-pigs. Inflammatory proteins (high-mobility group box 1 [HMGB1] or tumor necrosis factor alpha (TNF-α) were injected via a micro-infusion catheter into the vessel wall. All lesions were assessed with angiography and optical coherence tomography and all tissues were harvested for histological evaluation.

**Results:**

Intima/plaque area was significantly higher in the HMGB1- and TNF-α-injected groups compared to the saline-injected group (p = 0.002). CD68 antibody detection and polarization of M1 macrophages significantly increased in the inflammatory protein-injected groups (p<0.001). In addition, advanced atherosclerotic plaques were observed more in the inflammatory protein-injected groups compared with the control upon histologic evaluation.

**Conclusion:**

Direct injection of inflammatory proteins was associated with acceleration of atherosclerotic plaque formation with M1 macrophage polarization. Therefore, direct delivery of inflammatory proteins may induce a pro-inflammatory response, providing a possible strategy for development of an advanced atherosclerotic large-animal model in a relatively short time period.

## Introduction

Atherosclerosis is the primary cause of coronary and cerebrovascular disease, which is the leading cause of death worldwide [1]. The advanced atherosclerotic process is a result of a complex inflammatory and immune response [2]. High levels of low-density lipoprotein-cholesterol are associated with the accumulation of oxidized low-density lipoprotein in the vascular inner wall, which can trigger the formation of monocytes and their differentiation into macrophages in the arterial wall [3]. Macrophages play a pivotal role in the development, progression, and rupture of atherosclerotic plaques.

Plaque stability, rather than absolute size, determines whether atherosclerosis is clinically silent or pathogenic because unstable plaques can rupture, producing vessel-occluding thrombosis and end-organ damage. Stable plaques have a relatively thick fibrous cap, which consists largely of vascular smooth muscle cells (SMC) and extracellular matrix components, partitioning soluble clotting factors in the blood from thrombogenic molecules in the plaque [4]. In advanced atherosclerotic disease, plaques destabilize when elevated local matrix metalloproteinase degrades and thins the fibrous cap, increasing the risk of lesion rupture and subsequent thrombosis [5].

The macrophages in an atherosclerotic plaque can be categorized into the M1/M2 phenotype. Polarization of M1 macrophages results in progression and enlargement of an atherosclerotic plaque. The reversal to M2 macrophages can affect plaque regression via anti-inflammatory effects [6, 7]. Although several atherosclerotic animal models have been applied in experimental studies, a swine model of advanced atheromatous plaques is not yet practically available [8–10]. In our recent study, we demonstrate that direct injection of inflammatory proteins (high-mobility group box 1 [HMGB1] and tumor necrosis factor alpha [TNF-α]) to the vessel wall accelerates the inflammatory process and the development of advanced atherosclerotic plaques in a rabbit model [11].

The swine cardiovascular system is similar to that of human coronary and peripheral arteries in terms of vessel size and structure, making them an appropriate model for investigation of pathophysiologic mechanisms and application of devices in a preclinical setting [12, 13]. However, the swine model can be influenced by several genetic and environmental factors, which may be related to variable formation of atheromatous plaques, presenting a critical hurdle for establishment of an atherosclerotic model. In order to overcome these limitations and develop a consistent swine animal model, we directly injected pro-inflammatory material into the vessels as a reasonable solution for enhancing the pro-inflammatory reaction and inducing consistent atherosclerotic plaques.

## Materials and methods

### Animal ethics statement

The study protocol was approved by the local institutional animal care and use committee (Medi Kinetics, MK-IACUC: 130222–001). All animals received humane care in compliance with the Animal Welfare Act and the "Principles of Laboratory Animal Care" formulated by the Institute of Laboratory Animal Resources (National Research Council, NIH Publication No. 85–23, revised 1996). All mini-pigs were allowed free access to food and water during the study. The mini-pigs were allowed to acclimate to their environment for at least 7 days, and were then offered a high-cholesterol diet (2% cholesterol, Scientific Animal Food & Engineering, France). After a 1-week pretreatment with antibiotics and analgesics, anesthesia was induced by intramuscular injection of an appropriate mixture of Zoletil (0.2 ml/kg, Zoletil^®^, Virvac) and Rompun (0.1 ml/kg, Rompun^®^, Bayer), then maintained with 1.5% isoflurane (Forane^®^, JW Pharm, Seoul, Korea) and oxygen. Heparin (200 U/kg) was injected to maintain an activated clotting time >250 s before catheterization.

### Surgical procedures

A total of eight male mini-pigs (30–35 kg) were used in the study. The experimental protocol is illustrated (S1 Fig). Access to the coronary and iliac arteries was obtained through the carotid artery, using a surgical technique. Based on the quantitative coronary or femoral angiography, an oversized balloon inflation size of 15 mm, with a balloon:vessel ratio of 1.3:1.0, was applied two times for 30 seconds at each artery. The location was based on the branch of the coronary or femoral vessels found through angiography. After six weeks following the balloon injury, injection of saline with micro-infusion catheter and follow-up. Six weeks following the balloon injury, proteins or saline were delivered to the coronary and iliac arteries using Cricket^™^ and Bullfrog^™^ Micro-Infusion catheters (Mercator Medsystems, San Leandro, CA, USA). Angiography and optical coherence tomography (OCT) was then performed. All animals received aspirin (10 mg/kg) and clopidogrel (75 mg/day) following the procedure. Animals were sacrificed using the potassium chloride (KCL) during respiratory anesthesia, 4 weeks after the procedure, and coronary and femoral arteries were harvested.

### Injection of inflammatory proteins

The coronary and iliac arteries (32 total) from the 8 mini-pigs were divided and blindly and randomly assigned to 3 groups: saline injection (n = 11), high-mobility group box 1 (HMGB1, A&RT, Daejeon, Korea, n = 11), and tissue necrosis factor-alpha (TNF-α) (Prospec, Ness-Ziona, Israel, n = 10). After six weeks following the balloon injury, a balloon was inflated at the previous site of injury and the vessel wall penetrated by the Micro-Infusion catheter. Whole occlusion after balloon inflation and the shape of the needle was reconfirmed by angiography. The infusion catheter injected 200 μl saline, HMGB1, or TNF-α (proteins were at a concentration of 20 μg/200 μl saline) into the vessel wall. Each coronary and femoral artery received 2 treatments, and the injection was repeated 2 times within the injured vessel site to achieve the target dose of 400 μl with 60 seconds’ interval. One experienced operator for micro-infusion catheter performed this entire procedure. Angiography and OCT were performed immediately at the time of the procedure and 4 weeks following the procedure.

### Cell treatment

RAW 264.7 macrophages were maintained at 37°C in incubators containing an atmosphere of 5% CO_2_. Cells were cultured in RPMI-1640 medium supplemented with 10% fetal bovine serum, 1% penicillin, 1% MEM non-essential amino acid and 0.1% 2-mercaptoethanol. RAW 264.7 macrophages were incubated in 4 well culture slide. The drugs were treated at the following concentrations; HMGB1 0.5 μg/ml, TNF-α 0.1 μg/ml, LPS 0.1 μg/ml. Experiments were conducted after 15 hours of treatment with drugs.

### Morphological analysis

At the time of sacrifice, the 5mm-samples of total 15mm extracted from coronary and femoral vessels were fixed in 10% normal buffered formalin overnight before histological processing. Paraffin sections were cut on a microtome RM2235 (Leica, Wetzlar, Germany) at 4 μm, mounted on microscope slides (Superfrost Plus, Fisherbrand, Fisher Scientific, Waltham, MA, USA), and stained with hematoxylin and eosin (H&E), Masson’s trichrome, Movat’s pentachrome, and Sirius red stain for collagens. All pathologic slides were reviewed by one pathologist (S.K.) who performed the histomorphometric analysis.

1. Media area = External elastic lamina (EEL)–Internal elastic lamina (IEL)
2. Intima area = IEL-Lumen
3. Plaque area = Media + Intima

### Immunohistochemical (IHC) analysis

Inflammation and macrophage expression were evaluated by IHC analysis. Sections were immunostained with an antibody against the inflammatory markers, the receptor for advanced glycation end products (RAGE, LSBio, Seattle, WA, USA), HMGB1 (NOVUS, Littleton, CO, USA), or TNF-α (ABCAM, Cambridge, United Kingdom), and the macrophage markers CD68 (Thermo Scientific, Long Beach, NY, USA), CD47 (LSBio), and CD206 (Biorbyt, Cambridge, United Kingdom) at 4°C overnight. The primary antibody was directed against the antigen using a peroxidase-based kit (LSAB, DAKO, Glostrup, Denmark), and visualized by 3,3′-diaminobenzidine (DAB, DAKO) substrate with enhancer. The sections were subsequently counterstained with hematoxylin (DAKO). Changes in smooth muscle actin (SMA) were also evaluated with anti-α-SMA (ABCAM), following a general IHC protocol. Digital images of the vessels were scanned using a Leica SCN400, and histomorphometry analysis performed using LAS 4.2 software.

### Confocal immunofluorescence microscopy

Vessel tissue sections were micro cut at room temperature and then deparaffinized through the dewatering process. Subsequently, the sections were triple immunostained with anti-CD68 (Santa Cruz Biotechnologies, Santa Cruz, CA, USA), inducible nitric oxide synthase (iNOS, Santa Cruz Biotechnologies) for M1 macrophages, and arginase-1 (BD Biosciences, Franklin Lakes, NJ, USA) for M2 macrophages at 4°C overnight. The sections were washed for 10 min in 1% phosphate-buffered saline (PBS), and then secondarily labeled with FITC-conjugated donkey anti-goat IgG, PE-conjugated goat anti-rabbit IgG, and TR-conjugated goat anti-mouse IgG (Santa Cruz Biotechnologies), respectively, for 1 hour in the dark at room temperature. The sections were washed in PBS for 10 minutes, mounted with Fluoroshield with 4′,6-diamidino-2-phenylindole (DAPI, ImmunoBioscience, Mukilteo, WA, USA), and stored in the dark at 4°C until confocal microscopy was performed with an LSM 700 system (Carl Zeiss, Germany). The quantification of CD68, iNOS and Arg1 were analyzed ZEN software. Then we calculated the co-stained area of each iNOS, Arg-1 in CD68 expression within three representative region of interest rectangles per staining slide (n = 4 vessels/group).

### Terminal deoxynucleotidyl transferase (TdT)-mediated dutp nick end labeling (TUNEL) double-staining for apoptosis

Sections were stained with anti-BAX (Cell Signaling, Danvers, MA, USA), anti-cleaved caspase-3 (ABCAM), anti-α-SMA (ABCAM), or CD68 (Thermo Scientific) at room temperature for 2 hours. TUNEL solution and secondary antibodies (PE-conjugated goat anti-rabbit IgG and PE-conjugated goat anti-mouse IgG [Santa Cruz Biotechnologies]) were then added and sections incubated for 1 hour at 37°C in the dark. TUNEL assays were performed using the DeadEnd Fluorometric TUNEL System (Promega, Madison, WI, USA) according to the manufacturer’s instructions. Sections were mounted using Fluoroshield with DAPI and confocal microscopy was performed with a LSM 700 system (Carl Zeiss).

### Angiographical analysis

Quantitative coronary and femoral angiography analysis was performed before protein injection, following protein injection, and at the terminal procedure using the QAngio XA 7.1 Medis System (Medis Medical Imaging Systems, Leiden, Netherlands). All angiograms were analyzed in an independent core laboratory (Cardiovascular Research Center, Seoul, Korea) using the same analysis method as in our previous study [11]. Using the guiding catheter for magnification-calibration, the reference vessel diameter (RD) and minimum luminal diameter (MLD) were measured from diastolic frames in a single, matched view showing the smallest MLD. Percent diameter of stenosis was calculated according to the following formula: [1 - (lumen diameter/mean reference vessel diameter)] * 100.

### OCT analysis

OCT images were obtained at the terminal procedure using the C7-XR imaging systems (LightLab Imaging, Inc., St. Jude Medical, St. Paul, MN, USA) with the contrast agent at a 1:1 ratio with saline. Data were constantly acquired and stored digitally for later analysis, as described previously [11]. All OCT data was analyzed in an independent core laboratory and characterized and classified according to the following imaging features: 1) fibrous (homogeneous, high backscattering region); or 2) lipid (low-signal region with diffuse border) [14]. Macrophage infiltration within the lesion was characterized by increased signal intensity within the lesion accompanied by the heterogeneous backward shadows and classified as per the American Heart Association criteria [15].

### Blood analysis

Blood samples were obtained from the carotid of mini-pigs fasted overnight. Samples were collected prior to the start of the study, at the end of the high-cholesterol diet, and at the 11-week follow-up. Serum levels of total cholesterol, triglyceride, and high-density lipoprotein cholesterol were measured by DRI-CHEM 4000i (Fujifilm, Tokyo, Japan).

### Reverse transcription-PCR

Total RNA was isolated from the mini-pig iliac artery (5mm length) using QIAzol-reagent (QIAGEN, Hilden, Germany), and complementary DNA (cDNA) was synthesized using Quantitect Reverse Transcription (QIAGEN). The cDNA was amplified using AccuPower PCR Premix (Bioneer, Daejeon, Korea). The following primer sequences were used: RAGE, forward 5′-AAG CTT GGA AGG TCC TGA CT-3′ and reverse 5′-ACT TGG TCT CCT TTC CGT TC-3′; HMGB1, forward 5′-CCA TTG GTG ATG TTG CAA AG-3′ and reverse 5′-AGC CTT GAC GAC TCC CTT TT-3′; TNF-α, forward 5′-CCA CCA ACG TTT TCC TCA CT-3′ and reverse 5′-CCA AAA TAG ACC TGC CCA GA-3′; glyceraldehyde 3-phosphate dehydrogenase (GAPDH), forward 5′-GTC GGT TGT GGA TCT GAC CT-3′ and reverse 5′-AGC TTG ACG AAG TGG TCG TT-3′. Relative mRNA levels were determined by comparison to GAPDH mRNA. All PCR products were separated by electrophoresis on 1.5% agarose gels. Size was compared to a 100-bp DNA ladder (DYNE Bio, Seongnam, Korea), and PCR products were visualized using Loading STAR (DYNE Bio).

### Western blot analysis

Mini-pig iliac artery (5mm length) was lysed with radioimmunoprecipitation assay (RIPA) buffer (Biosesang, Seongnam, Korea) containing a cOmplete Mini, ethylenediaminetetraacetic acid (EDTA)-free protease inhibitor cocktail (Roche, Basel, Switzerland). The protein samples were separated by a sodium dodecyl sulfate polyacrylamide gel electrophoresis (SDS-PAGE) and then electrotransferred to Immuno-Blot^®^ PVDF membrane (Bio-Rad, Hercules, CA, USA). Membranes were blocked with 5% skim milk (Noble Bio, Hwaseong, Korea) in Tris-buffered saline-Tween 20 (TBS-T) for 1 hour at room temperature. Membranes were incubated with primary antibodies overnight, washed with TBS-T, incubated with horseradish peroxidase-conjugated secondary antibody for 1 hour, and then subjected to enhanced chemiluminescence (ECL) detection. All blots were probed using GAPDH as a loading control on the same membrane. Densitometry analysis was performed with Image J software (National Institutes of Health, Bethesda, MD, USA).

### Statistical analysis

Data were expressed as mean ± SEM. Statistical analyses were performed using SPSS v20.0 (SPSS Inc., Chicago, IL, USA). Continuous variables were compared using one-way analysis of variance (ANOVA). P-values less than 0.05 were considered statistically significant.

## Results

### Histomorphometric analysis and cholesterol level

**Fig 1:**
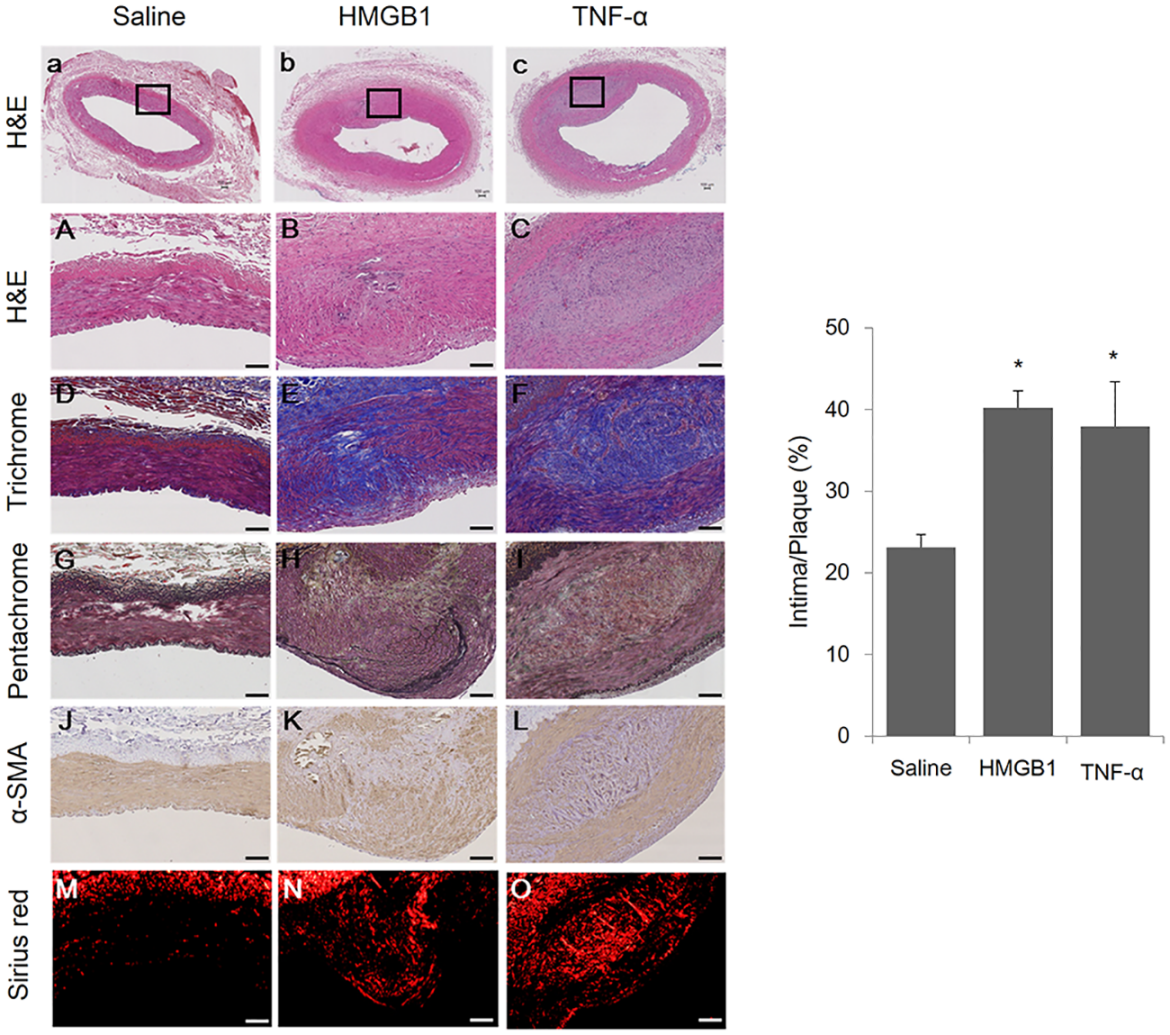
Analysis of morphologic changes in arteries of mini-pigs with induced atherosclerosis. The α-SMA content of the arteries was detected by IHC staining of α-SMA; the collagen content of the plaques is represented by Sirius red staining visualized under polarized light. Representative images of plaques injected with either saline (n = 11), HMGB1 (n = 11), and TNF-α (n = 10) as indicated and stained with hematoxylin & eosin (H&E) (a-c [20× of A-C]), Masson’s trichrome (D-F), Masson’s pentachrome (G-I), α-SMA (J-L), and Sirius red (M-O) (100×). Black boxes in a, b, and c represent the areas further magnified in A-O corresponding to their respective columns. IHC area was calculated as (intima/plaque) × 100, %. Scale bars represent 100 μm. The intima-plaque ratio data are represented as the mean±SEM. *p<0.05, compared with the saline group.

The target lesions (n = 32) of coronary and femoral arteries were harvested from 8 mini-pigs at 10 weeks following injection of inflammatory proteins (saline [n = 11], HMGB1 [n = 11], and TNF-α [n = 10]). The incidence of advanced plaque type, as determined by the American Heart Association criteria [16], was higher in the inflammatory protein groups compared to the saline group (Fig 1).

Furthermore, the intima/plaque area ratio significantly increased in the HMGB1 and TNF-α groups compared to the saline group (HMGB1 group: 40.2±2.1%, TNF-α group: 37.9±5.5%, saline group: 23.1±1.6%, p = 0.002) (S1 Table). Histomorphometric analysis using Sirius red and alpha-smooth muscle actin (α-SMA) showed that collagen and SMC deposition in the media layer were increased in the HMGB1- and TNF-α-injected groups compared to the saline-injected group.

The mean baseline level of total cholesterol was 44.8±1.0 mg/dl, and this level significantly increased to 366.85±16.3 mg/dl after initiation of a 2% cholesterol diet. Low-density lipoprotein-cholesterol, triglycerides, and high-density lipoprotein-cholesterol increased to 306.1±16.9 mg/dl, 7.0±1.1 mg/dl, and 59.3±2.1 mg/dl at 7 weeks, respectively.

### Inflammatory marker expression and macrophage infiltration within plaques

**Fig 2:**
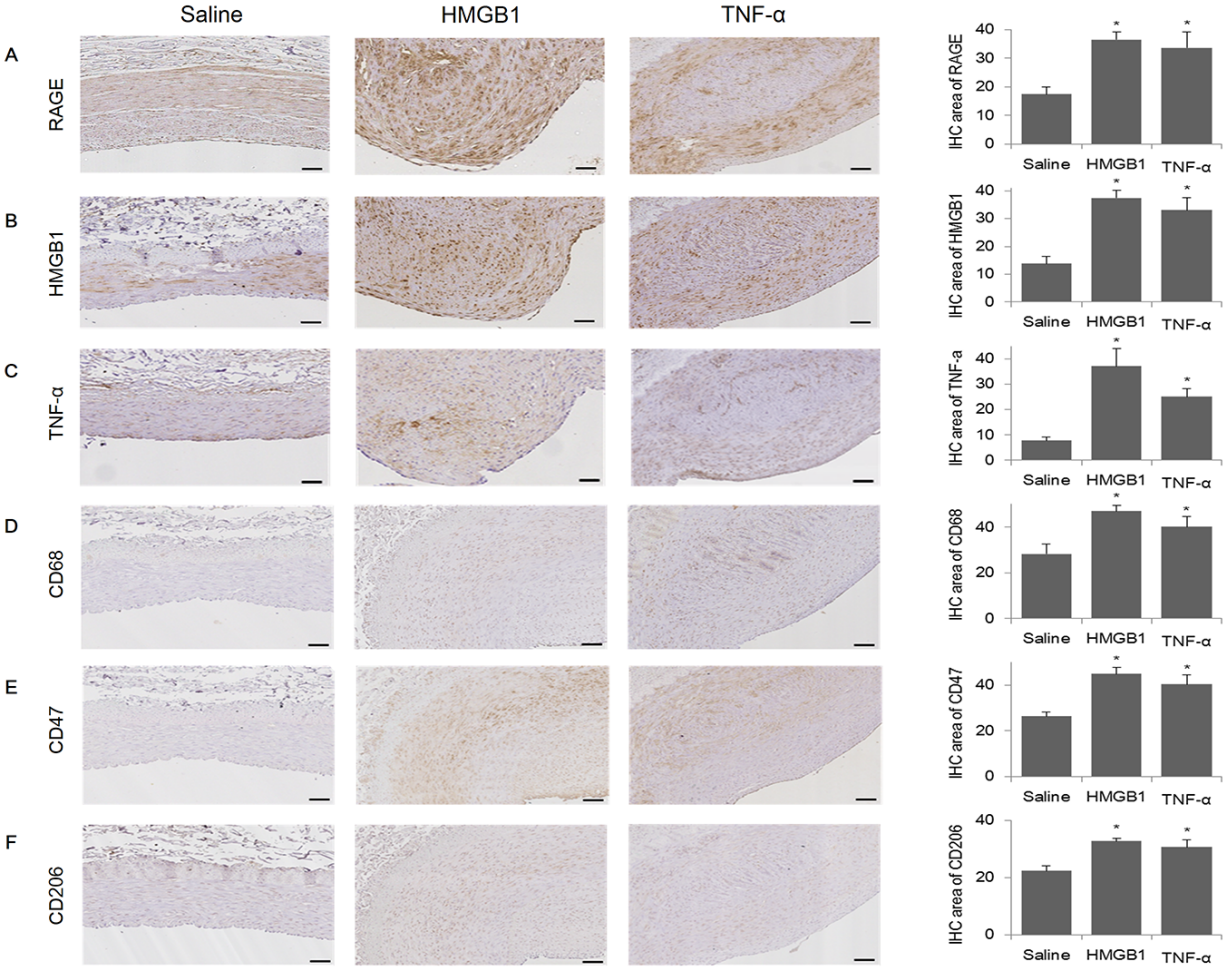
IHC analysis of the degree of inflammation and macrophages present in the arteries of mini-pigs with induced atherosclerosis. Inflammation and macrophages present in arterial plaques was detected by Immunohistochemical (IHC) staining for RAGE, HMGB1, TNF-α, CD68, CD47, or CD206 in the saline (n = 11), HMGB1 (n = 11), and TNF-α (n = 10) groups. Representative images of IHC staining of RAGE (A), HMGB1 (B), TNF-α (C), CD68 (D), CD47 (E), and CD206 (F) in the mini-pig artery (amplification 100×). IHC area was calculated as (intima/plaque) × 100, %. Scale bars represent 100 μm. Quantitative data are represented as the mean±SEM. *p<0.05, compared with the saline group.

The expression of receptors for advanced glycation end-products (RAGE) within the plaques significantly increased in HMGB1 and TNF-α groups compared with the saline group (36.6±2.7% and 33.6±5.6%, respectively, compared with 17.4±2.5%, p = 0.003) as assessed by immunohistochemistry (IHC). The expression of HMGB1 (HMGB1, 37.5±2.7% and TNF-α, 33.0±4.5% compared with saline, 13.8±2.8%, p<0.001) and TNF-α (HMGB1, 37.1±7.1% and TNF-α, 25.1±3.0% compared with saline, 7.8±1.4%, p<0.001) within the plaques were also significantly increased in HMGB1 and TNF-α groups compared with the saline group (Fig 2).

Reverse transcription-PCR showed that RAGE and TNF-α mRNA expression was significantly higher in the HMGB1 group compared with the saline group (S2 Fig). Accordingly, RAGE and TNF-α protein expression were significantly higher in the inflammatory protein-injected groups compared with the saline group (S3 Fig).

Macrophage infiltration is a well-known histologic feature found in advanced atherosclerotic plaques. Macrophage infiltration, as demonstrated by CD68 IHC staining within plaques, was significantly increased in the HMGB1 and TNF-α groups compared with the saline group (46.9±2.7% and 40.2±4.4%, respectively, compared with 28.2±4.3%, p = 0.005). In addition, staining with the recently identified macrophage markers CD47 (HMGB1, 45.0±2.9% and TNF-α, 40.4±4.0% compared with saline, 26.4±1.9%, p = 0.001) and CD206 (HMGB1, 32.8±1.1% and TNF-α, 30.7±2.5% compared with saline, 22.4±1.7%, p = 0.002) was significantly increased in the inflammatory protein-injected groups compared with the saline group (Fig 2).

### Macrophage polarization analysis (M1 vs. M2) in plaques

**Fig 3:**
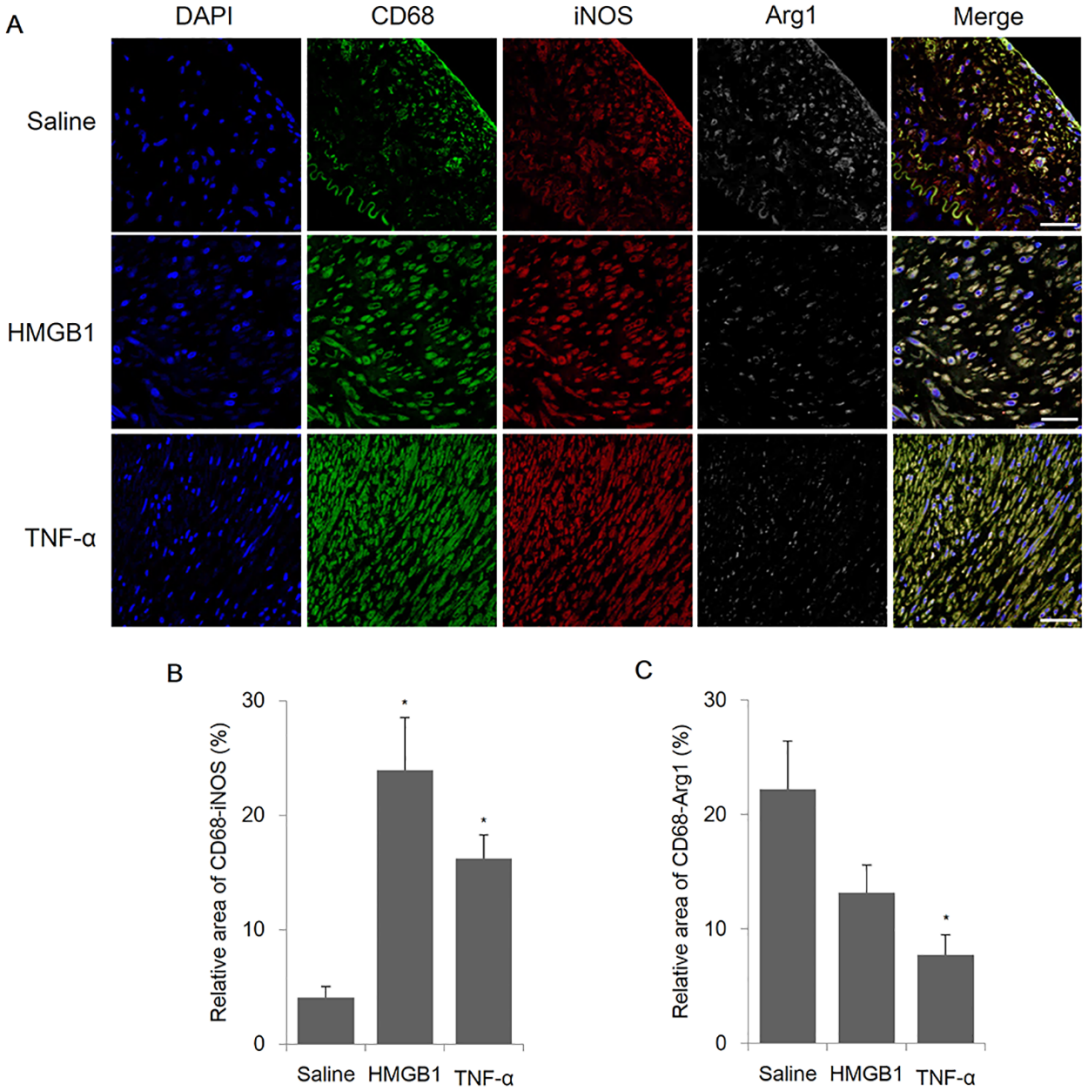
IF analysis of macrophage infiltration in the arteries of mini-pigs with induced atherosclerosis. The macrophage content of the plaques was detected by immunofluorescence (IF) using CD68, inducible nitric oxide synthase (iNOS; M1), and arginase-1 (Arg1; M2) antibodies. A) Representative images of M1/M2 macrophage immunofluorescence in the mini-pig artery of the saline, HMGB1, and TNF-α groups (n = 4 vessels/group) (amplification 400×). B) Measurement of the co-expression area of CD68-iNOS. C) Measurement of the co-expression area of CD68-Arg1. Relative area measurements were determined using a Zeiss LSM 700. Scale bars represent 100 μm. Data are represented as the mean±SEM. *p<0.05, compared with the saline group.

Macrophage polarization, particularly to M1 rather than M2, induces advanced atherosclerosis within plaques. Immunofluorescence analysis indicated that the pro-inflammatory macrophage marker of M1 (inducible nitric oxide synthase) significantly increased in HMGB1 and TNF-α groups compared with the saline group (23.9±4.6% and 16.2±2.1%, respectively, compared with 4.1±1.0%, p<0.001). Expression of the anti-inflammatory macrophage, M2, marker (arginase-1) significantly decreased in the TNF-α group compared with the saline group (7.7±1.7% compared with 22.2±4.2%, p = 0.212) within plaques (Fig 3). These findings were confirmed in vitro using macrophage cell line, RAW 264.7 (S4 Fig).

### Apoptosis of SMC and macrophages within plaques

**Fig 4:**
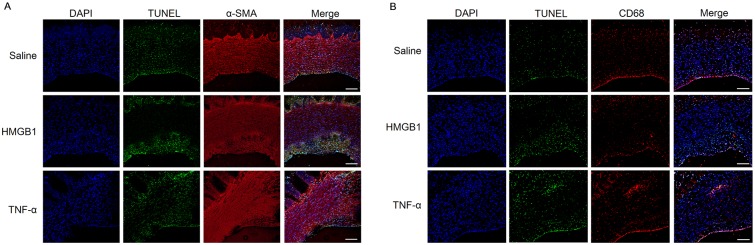
Determination of apoptosis in the arterial plaques of mini-pigs with induced atherosclerosis using the TUNEL assay and co-IF stain of α-SMA and CD68. The apoptosis content of the plaques was detected by terminal deoxynucleotidyl transferase (TdT)-mediated dUTP nick end labeling (TUNEL) assay and immunofluorescence (IF) using alpha-smooth muscle actin (α-SMA) and CD68 antibodies. Representative images of mini-pig arteries in the saline (n = 11), HMGB1 (n = 11), and TNF-α (n = 10) groups stained with (A) TUNEL and α-SMA or (B) TUNEL and CD68 (amplification 100×). Digital images of the vessels were scanned using a Zeiss LSM 700. Scale bars represent 100 μm.

SMC and macrophage apoptosis is present in advanced atherosclerotic plaques. Cell apoptosis was confirmed using the terminal deoxynucleotidyl transferase (TdT)-mediated dUTP nick end labeling (TUNEL) assay. TUNEL-positive cells were detected in both intima and media layers of SMC, as identified by SMA-positive and CD68-positive areas. HMGB1- and TNF-α-injected groups demonstrated further increased expression of apoptotic cells in the vessel area, particularly in the intima layer (Fig 4).

Moreover, large numbers of cells expressed pro-apoptotic protein; Bax and cleaved-caspase-3 were observed in these areas where apoptosis was detected via the TUNEL assay (S5 Fig).

### Optical coherence tomography (OCT) and quantitative imaging analysis

**Fig 5:**
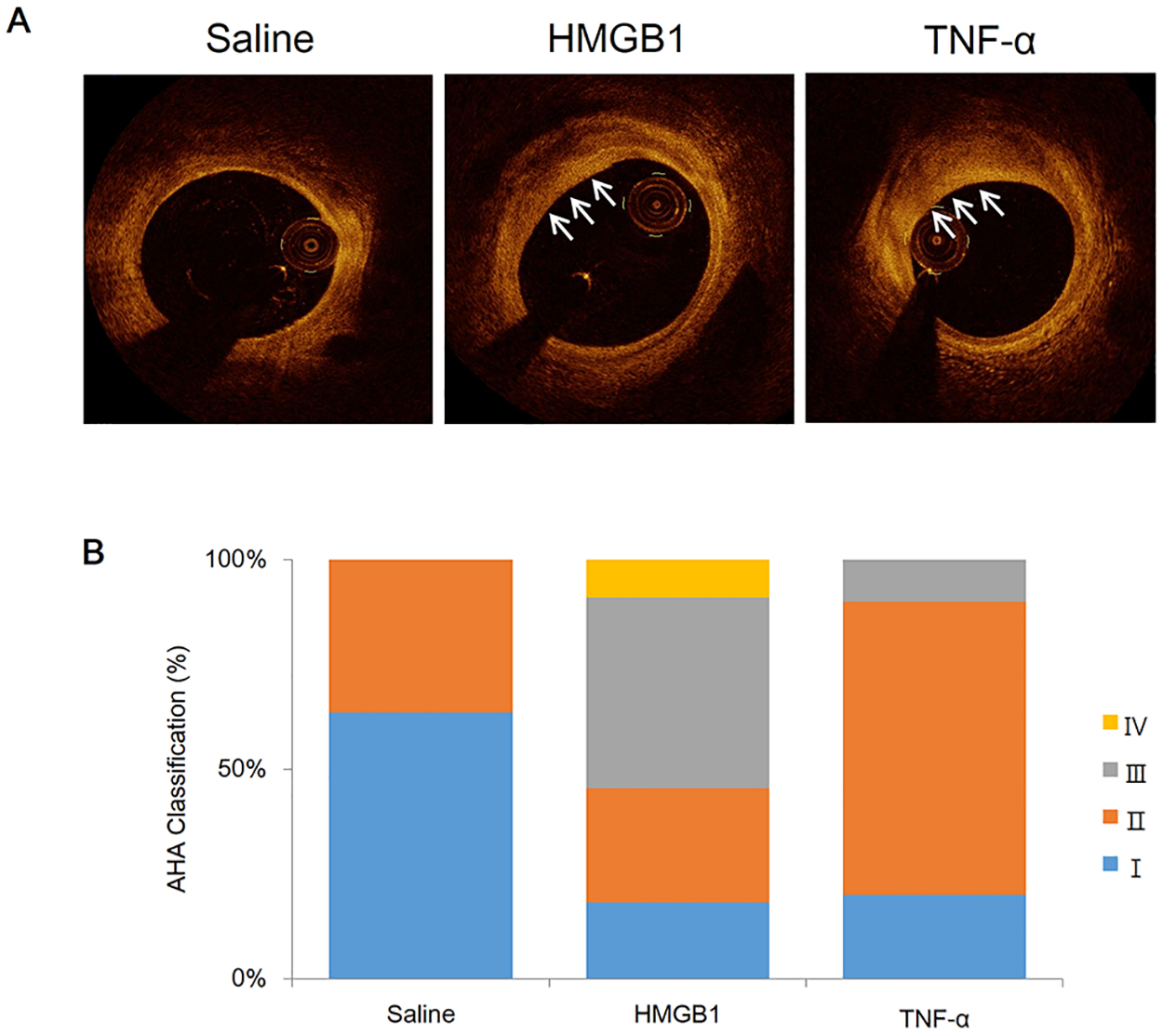
OCT of atheromatous characteristics and histologic classification based on AHA criteria. (A) Optical coherence tomography (OCT) images of atheromatous characteristics of the arteries of mini-pigs with induced atherosclerosis from the saline (n = 11), HMGB1 (n = 11), and TNF-α (n = 10) groups. White arrows indicate location of plaques. (B) Histologic classification of plaques from each group according to the American Heart Association (AHA) criteria. Plaque characteristics were categorized into early (type I [blue], II [orange], and III [grey]) and advanced (type IV [yellow]).

Fig 5A displays representative OCT images of lipid and macrophage accumulation in plaques of inflammatory protein-injected groups, and Fig 5B shows the analysis of plaque classification by qualitative analysis.

The proportion of lipid-rich plaques differed among the groups (HMGB1 group: 9/11 [82%], TNF-α group: 5/10 [50%], saline group: 2/11 [18%]). The percent area stenosis significantly increased in HMGB1 and TNF-α groups compared with the saline group (46.4±1.2% and 42.6±3.1%, respectively, compared with 35.5±1.5%, p = 0.001). The percentage of diameter stenosis was significantly increased in HMGB1 and TNF-α groups compared with the saline group (30.5±1.9% and 31.7±2.5%, respectively, compared with 21.3±0.7%, p<0.001) (Table 1 and S2 Table).

**Table 1:** Morphological parameters in histology, optical coherence tomography, and quantitative angiography.

	Saline (N = 11)	HMGB1 (N = 11)	TNF-α (N = 10)
Histology			
Media, mm^2^	1.13±0.13	1.10±0.26	1.21±0.19
Intima, mm^2^	0.32±0.03	0.69±0.11*	0.79±0.18*
Plaque, mm^2^	1.45±0.15	1.80±0.36	2.00±0.28
I/P*100 (%)	23.11±1.56	40.28±1.78*	37.90±5.49*
OCT			
Vessel area, mm^2^	13.52±1.43	14.53±0.67	15.82±1.59
Lumen area, mm^2^	8.72±0.98	7.76±0.50	9.34±1.21
Plaque, mm^2^	4.81±0.56	6.77±0.27*	6.49±0.53*
Area stenosis, %	35.53±1.54	46.43±1.20*	42.64±3.08*
Lipid rich plaque, %	2/11 (18%)	9/11 (82%)	5/10 (50%)
QCA			
RD, mm	3.23±0.20	3.01±0.41	3.33±0.32
MLD, mm	2.55±0.18	2.11±0.33	2.32±0.27
DS, %	21.28±0.74	30.51±1.68*	31.71±2.47*

Values are n (%) or mean ± SEM. QA, quantitative angiography; OCT, optical coherence tomography; RD, reference diameter; MLD, minimal luminal diameter; DS, diameter stenosis; and I/P, intimal plaque ratio.

*p<0.05, compared with the Saline group

## Discussion

Here, we found that direct injection of inflammatory proteins was associated with acceleration of atherosclerotic plaque formation in swine coronary and peripheral arteries, as well as increased macrophage infiltration in atheromatous plaques and induced macrophage polarization to the pro-inflammatory M1 subtype. Furthermore, advanced atherosclerotic plaques were more frequently observed in the inflammatory protein-injected groups compared with the control via OCT and histologic analysis. Based on our observations, direct delivery of inflammatory proteins can be an alternative strategy to develop a reproducible large animal model with advanced atherosclerotic plaques.

The accepted method for inducing atherosclerosis is a combination of high-cholesterol diet-induced elevated blood cholesterol levels and causing damage to the endothelium of the intima using a balloon catheter. Additionally, we injected inflammatory proteins directly into the endothelium of the vessel, which is the site of HMGB1 and TNF-α participation in early mechanisms of atherosclerosis. Generally, long periods of a high-cholesterol diet are necessary to induce atherosclerosis in higher organisms (e.g., swine), resulting in increased cost. In our study, we provided the mini-pigs a high-cholesterol diet 1 week prior to endothelial damage, and 6 weeks following damage, for a total of 7 weeks to induce atherosclerosis. Through this method of injecting inflammatory proteins, we shortened the period during which a high-cholesterol diet was provided to induce atherosclerotic plaques, particularly advanced plaques.

Our study confirmed that the histological analysis value of the intima ratio significantly increased, and more inflammatory areas were detected in the inflammatory protein-injection group compared with the control, using several immunologic markers. We used two inflammatory proteins, HMGB1 and TNF-α, which induce early atherosclerosis. HMGB1 contributes to the pathogenesis of numerous chronic inflammatory and immunologic diseases in addition to atherosclerosis, such as chronic kidney disease, rheumatoid arthritis, and cancer pathogenesis [17, 18]. Released HMGB1 interacts with RAGE, one of the main signaling pathways triggered in sustained inflammatory related diseases [17], and TNF-α is a potent pro-inflammatory cytokine that can induce atherosclerosis [19, 20]. These data suggests that injection of inflammatory proteins may induce up-regulation of RAGE expression, and signal downstream activation of TNF-α and CD68, resulting in increased recruitment of macrophages through cytokine and cell adhesion molecule expression [21]. These results were consistent with our previous studies conducted in a rabbit model [11].

Atherosclerosis is an inflammatory disease; macrophages play a central role in all stages of atherosclerosis, from initiation, lesion expansion, and necrosis leading to rupture and the clinical manifestations of atherosclerosis, to regression and resolution of atherosclerotic lesions. Macrophage infiltration in atherosclerotic plaques was identified using CD68, a widely used macrophage marker. CD47 is a key anti-phagocytic molecule known to render malignant cells resistant to programmed cell removal, termed efferocytosis. Impaired efferocytosis plays a pathogenic role in cardiovascular disease, including atherosclerosis [22]. Recently published studies found that CD47-blocking antibodies could restore phagocytosis and prevent atherosclerosis [23]. CD206 (C-type mannose receptor 1) is expressed in various types of macrophages in cardiac, peritoneal, and adipose tissue, and is involved in clearing inflammatory molecules in the blood [24]. These markers are expressed in pathogens, apoptotic cells, and damaged tissues and molecules [25]. We confirmed the increased expression of these macrophage markers in inflammatory protein-injected groups. Taken together, these results demonstrated accelerated atherosclerosis in our large animal model.

Macrophages can be divided into two polarization systems (M1 and M2) according to their effect on inflammation (pro- vs. anti-inflammatory). In the pathophysiology of atherosclerosis, M1 macrophages have significant roles in plaque progression, monocyte recruitment into plaques, and vulnerable plaque development [26]. Moreover, M1 macrophages promote atherosclerosis by maintaining chronic inflammation, leading to foam cell formation [27]. In contrast, M2 macrophages cause regression of plaques, such as a reduction in plaque size, cholesterol content, and macrophage percentage, as well as a decreased inflammatory state in plaques [28]. We found increased macrophage infiltration in atherosclerotic plaques in our large animal model and an increased M1 detection ratio and decreased M2 detection ratio within atherosclerotic plaques using M1- or M2-specific immunofluorescence. Thus, we confirmed the polarization of macrophages to the M1 phenotype within atherosclerotic plaques, supporting the reliability of our swine model using inflammatory protein injection of HMGB1 and TNF-α.

The regulation of apoptosis can lead to disease development due to an accumulation of undead cells [29]. This mechanism is known to contribute to macrophage and vascular SMC (VSMC) apoptosis during atherosclerotic lesion development. Abnormal proliferation of VSMC promotes plaque formation, but VSMC in advanced plaques are beneficial because they can prevent rupture of the fibrous cap [30]. However, VSMC apoptosis and VSMC-derived macrophage-like cells may promote inflammation. In our study, apoptosis was observed more in the intima and media layer of the protein-injection groups compared to the controls. These results indicated that atherosclerosis was accelerated in the inflammatory protein-injection groups.

OCT has a high resolution and detects characteristics of atherosclerotic plaques in the clinical diagnosis of patients [14, 31]. Using OCT analysis, we confirmed that the HMGB1 and TNF-α groups had a higher accumulation of lipid-rich plaques compared with the saline group, indicating that the area stenosis was significantly increased in the protein-injection groups. Similar results were obtained for diameter stenosis through angiography analysis.

There were several limitations to our study. First, the experimental period was not sufficiently long to allow for the development of vulnerable features of plaques, such as a necrotic core or calcified plaques. Second, the mechanism of increased CD 68+ cells within plaque after inflammatory injection could not be investigated. Further studies are needed to determine the mechanism of accelerated atherosclerosis in inflammatory protein injected group. Despite these limitations, the current study clearly demonstrated that direct injection of inflammatory proteins into vessel walls accelerated atherosclerosis in a mini-pig model. We can apply this large-animal model to investigate the pathophysiologic process and then confirm the hypothesis in a small-animal model or in an *in vitro* study.

In conclusion, we found that direct injection of inflammatory proteins into the vessel wall accelerated the progression of atherosclerosis, increased macrophage infiltration, and induced macrophage differentiation to the M1 phenotype, which were morphological and morphometric features of accelerated atherosclerosis in a large-animal model. Accordingly, the inflammatory protein-injecting method for inducing advanced atherosclerosis can be conducted in a short experimental period with low cost.

## Supporting information

S1 Fig Experimental protocol. Schematic view of study timeline. Upon arrival to the animal facility, mini-pigs were started on a daily high-cholesterol diet for 7 weeks. After 1 week, balloon injury was induced, and at the end of 7 weeks, inflammatory proteins were injected. Body weight and plasmas were obtained prior to the high-cholesterol diet and again before sacrifice at 11 weeks. OCT was assessed at the beginning and end of the study. Animals were euthanized 4 weeks after the injection procedure, and iliac and coronary arteries harvested. Chol, cholesterol; OCT, optical coherence tomography.
(TIF)

S2 Fig Relative mRNA levels in the iliac arteries of mini-pigs with induced atherosclerosis. A, Reverse transcription-PCR analysis of RAGE, HMGB1, and TNF-α mRNA expression in the iliac artery from the saline, HMGB1, and TNF-α groups. B-D, Representative data of mRNA expression of RAGE, HMGB1, and TNF-α in the iliac artery from the saline (n = 7), HMGB1 (n = 3), and TNF-α (n = 6) groups (normalized to GAPDH). Data included in the bar graph are quantified ratios of the signals for RAGE, HMGB1, and TNF-α relative to GAPDH (fold increase). Data are presented as the mean±SEM. *p<0.05, compared with the saline group.
(TIF)

S3 Fig Relative protein levels in the iliac arteries of mini-pigs with induced atherosclerosis. Western blot analysis of RAGE, HMGB1, and TNF-α protein expression in the iliac artery from the saline (n = 7), HMGB1 (n = 3), and TNF-α (n = 6) groups. A-C, Representative western blot protein expression data of RAGE, HMGB1, and TNF-α in the iliac artery from the three groups (normalized to GAPDH). The bar graphs illustrate the quantified signals for RAGE, HMGB1, and TNF-α compared with GAPDH (fold increase). Data are presented as the mean±SEM. *p<0.05, compared with the saline group.
(TIF)

S4 Fig IF analysis of macrophage infiltration in the RAW264.7 macrophage cell. The macrophage content of the RAW264.7 was detected by immunofluorescence (IF) using CD68, inducible nitric oxide synthase (iNOS; M1), and arginase-1 (Arg1; M2) antibodies. A) Representative images of M1/M2 macrophage immunofluorescence in the RAW264.7 of the PBS, HMGB1 (0.5 μg/ml), TNF-α (0.1 μg/ml) and LPS (0.1 μg/ml) groups. Digital images of the cells were scanned using a Zeiss LSM 700. The amplification of 400×.
(TIF)

S5 Fig TUNEL assay and co-immunofluorescence of Bax and cleaved-Caspase-3. Apoptosis in mini-pig artery plaques from the saline, HMGB1, and TNF-α groups was detected by Determination of apoptosis in plaques using terminal deoxynucleotidyl transferase (TdT)-mediated dUTP nick end labeling (TUNEL) assay and immunofluorescence stain with Bax and cleaved-Caspase-3 antibodies. Representative images of mini-pig arteries from the saline, HMGB1, and TNF-α groups stained with (A) TUNEL and anti-Bax or (B) TUNEL and anti-cleaved-Caspase-3 (amplification 200×). Digital images of the vessels were scanned using a Zeiss LSM 700. Scale bars represent 100 μm.
(TIF)

S1 Table Coronary or femoral histology analysis raw data. I/P, intimal plaque ratio.
(PDF)

S2 Table Coronary or femoral quantitative angiography raw data. RD, reference diameter; MLD, minimal luminal diameter; DS, diameter stenosis.
(PDF)
