# Supplementary figures and images for: Macrophage polarization and acceleration of atherosclerotic plaques in a swine model
Source: PLoS One. 2018 Mar 21;13(3):e0193005. doi: 10.1371/journal.pone.0193005 (PMC5862407; doi:10.1371/journal.pone.0193005)

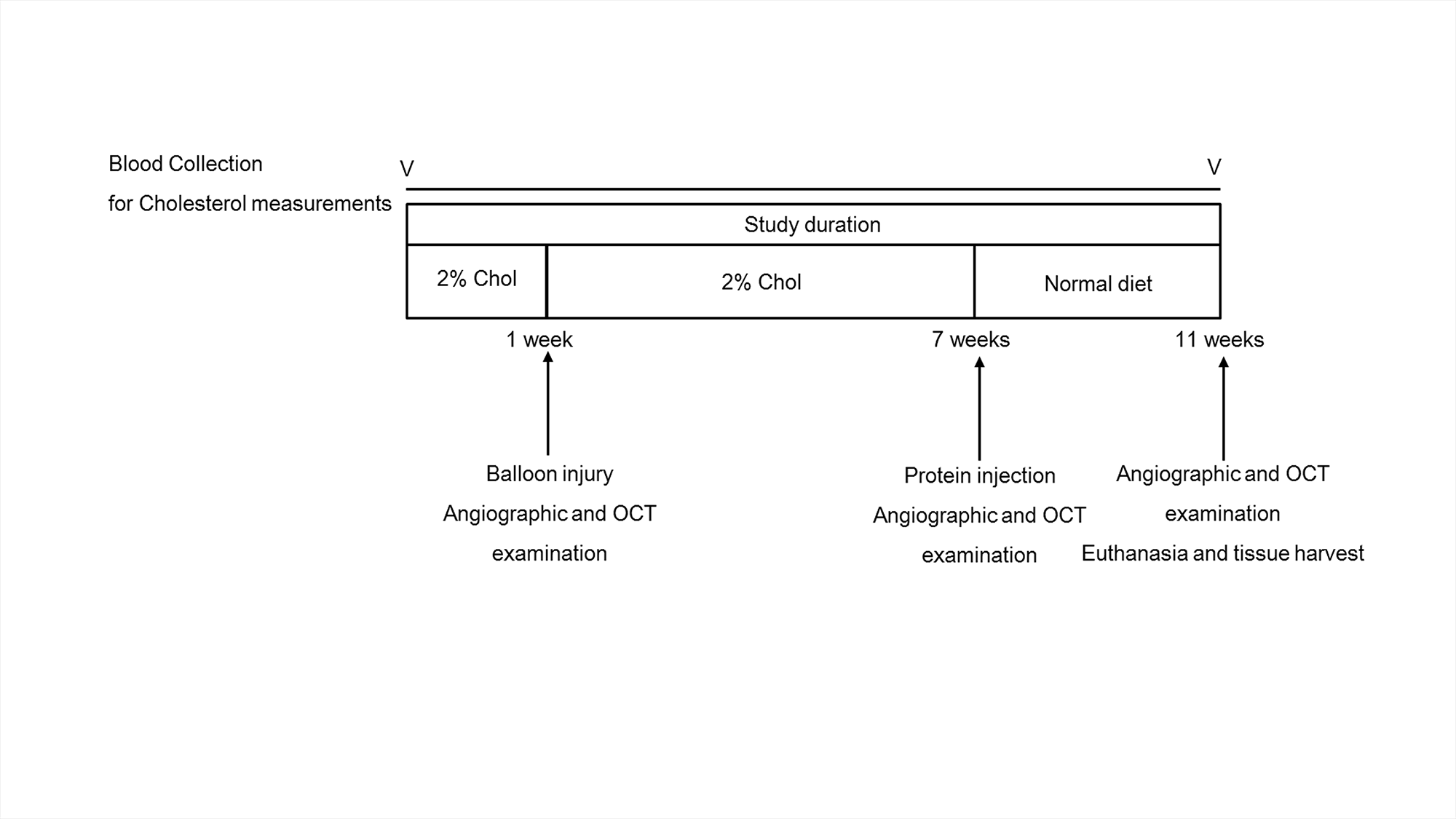

Supplement: S1 Fig — Schematic view of study timeline. Upon arrival to the animal facility, mini-pigs were started on a daily high-cholesterol diet for 7 weeks. After 1 week, balloon injury was induced, and at the end of 7 weeks, inflammatory proteins were injected. Body weight and plasmas were obtained prior to the high-cholesterol diet and again before sacrifice at 11 weeks. OCT was assessed at the beginning and end of the study. Animals were euthanized 4 weeks after the injection procedure, and iliac and coronary arteries harvested. Chol, cholesterol; OCT, optical coherence tomography. (TIF) [file pone.0193005.s001.tif]

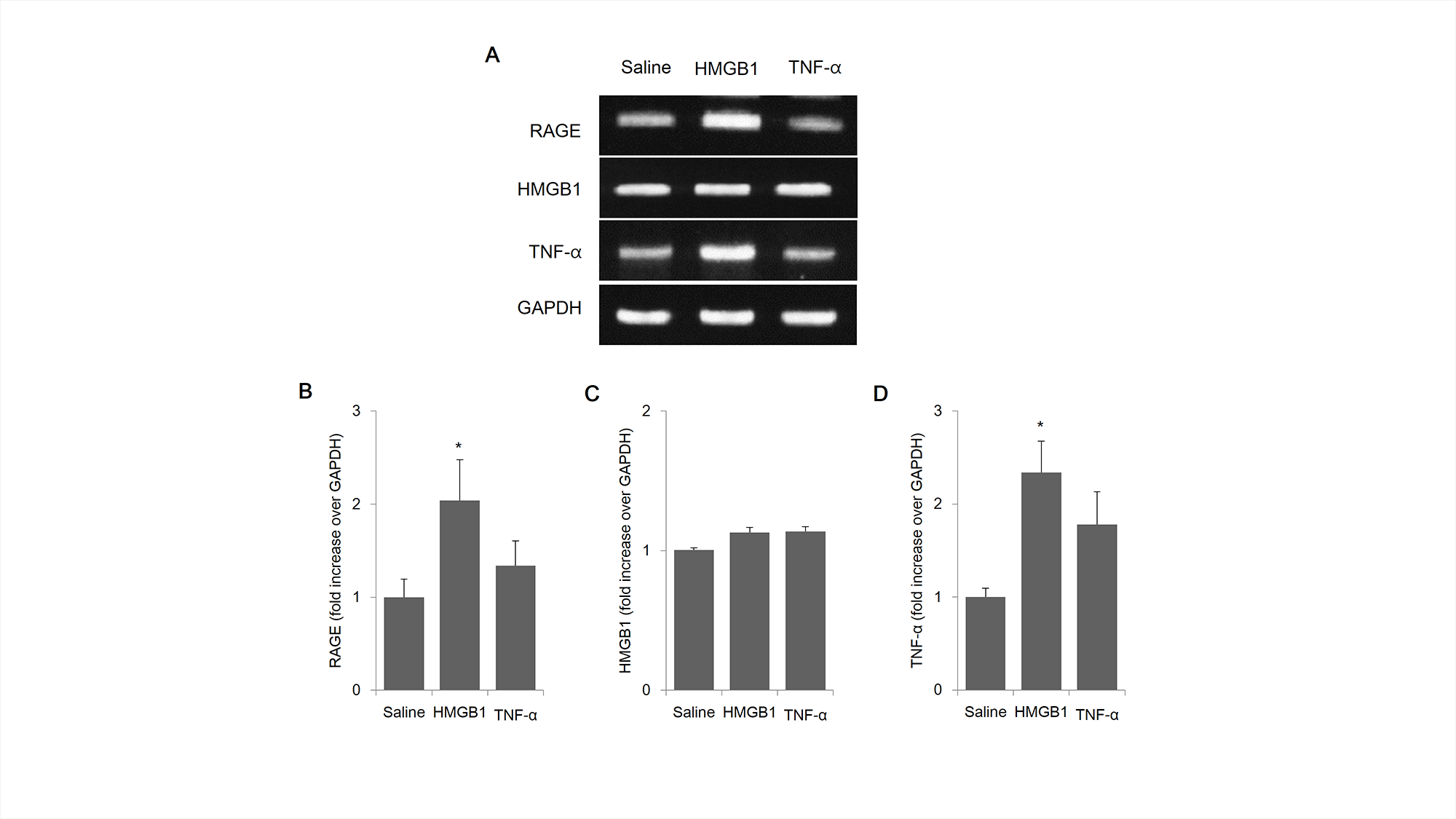

Supplement: S2 Fig — A, Reverse transcription-PCR analysis of RAGE, HMGB1, and TNF-α mRNA expression in the iliac artery from the saline, HMGB1, and TNF-α groups. B-D, Representative data of mRNA expression of RAGE, HMGB1, and TNF-α in the iliac artery from the saline (n = 7), HMGB1 (n = 3), and TNF-α (n = 6) groups (normalized to GAPDH). Data included in the bar graph are quantified ratios of the signals for RAGE, HMGB1, and TNF-α relative to GAPDH (fold increase). Data are presented as the mean±SEM. *p<0.05, compared with the saline group. (TIF) [file pone.0193005.s002.tif]

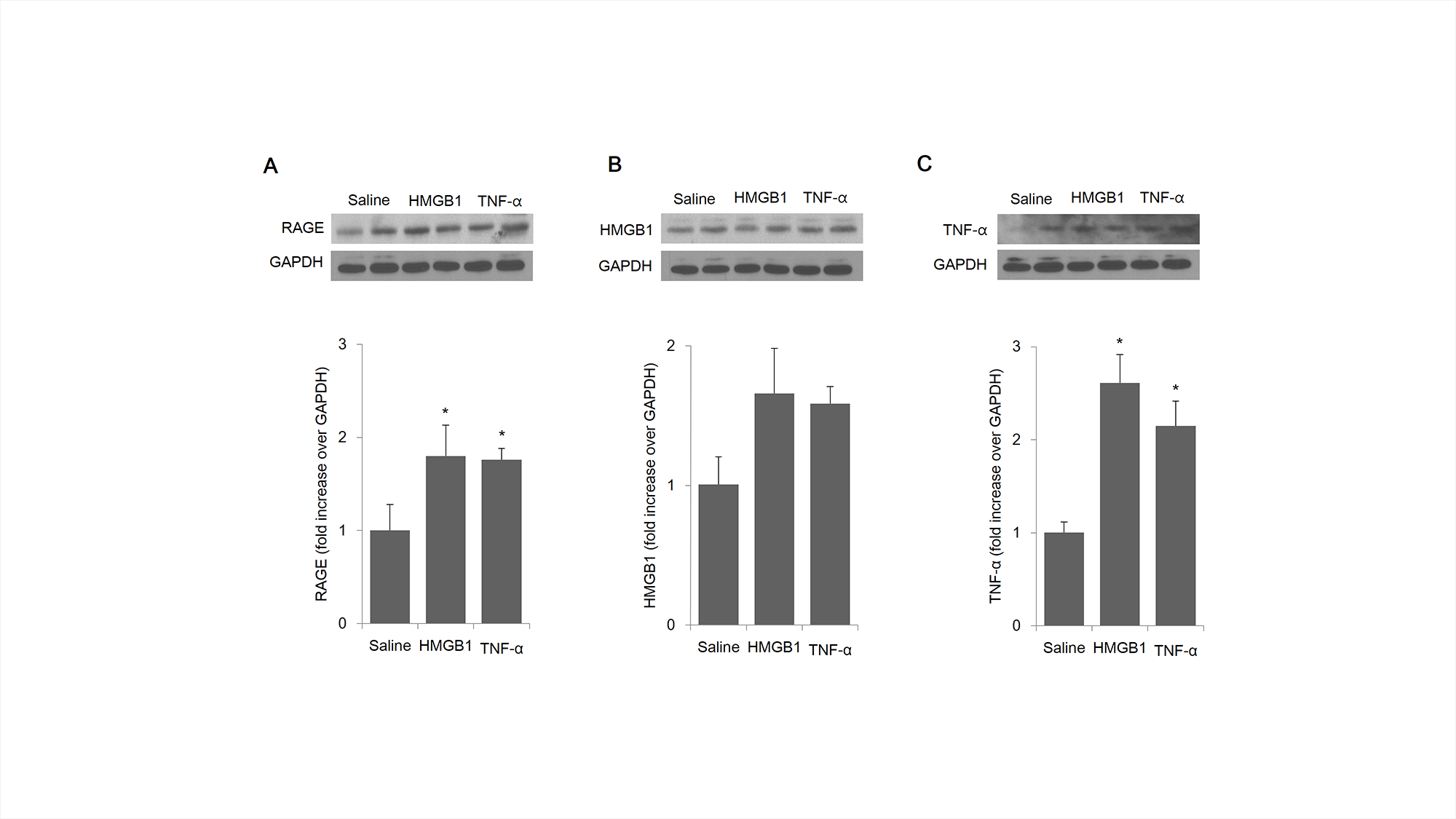

Supplement: S3 Fig — Western blot analysis of RAGE, HMGB1, and TNF-α protein expression in the iliac artery from the saline (n = 7), HMGB1 (n = 3), and TNF-α (n = 6) groups. A-C, Representative western blot protein expression data of RAGE, HMGB1, and TNF-α in the iliac artery from the three groups (normalized to GAPDH). The bar graphs illustrate the quantified signals for RAGE, HMGB1, and TNF-α compared with GAPDH (fold increase). Data are presented as the mean±SEM. *p<0.05, compared with the saline group. (TIF) [file pone.0193005.s003.tif]

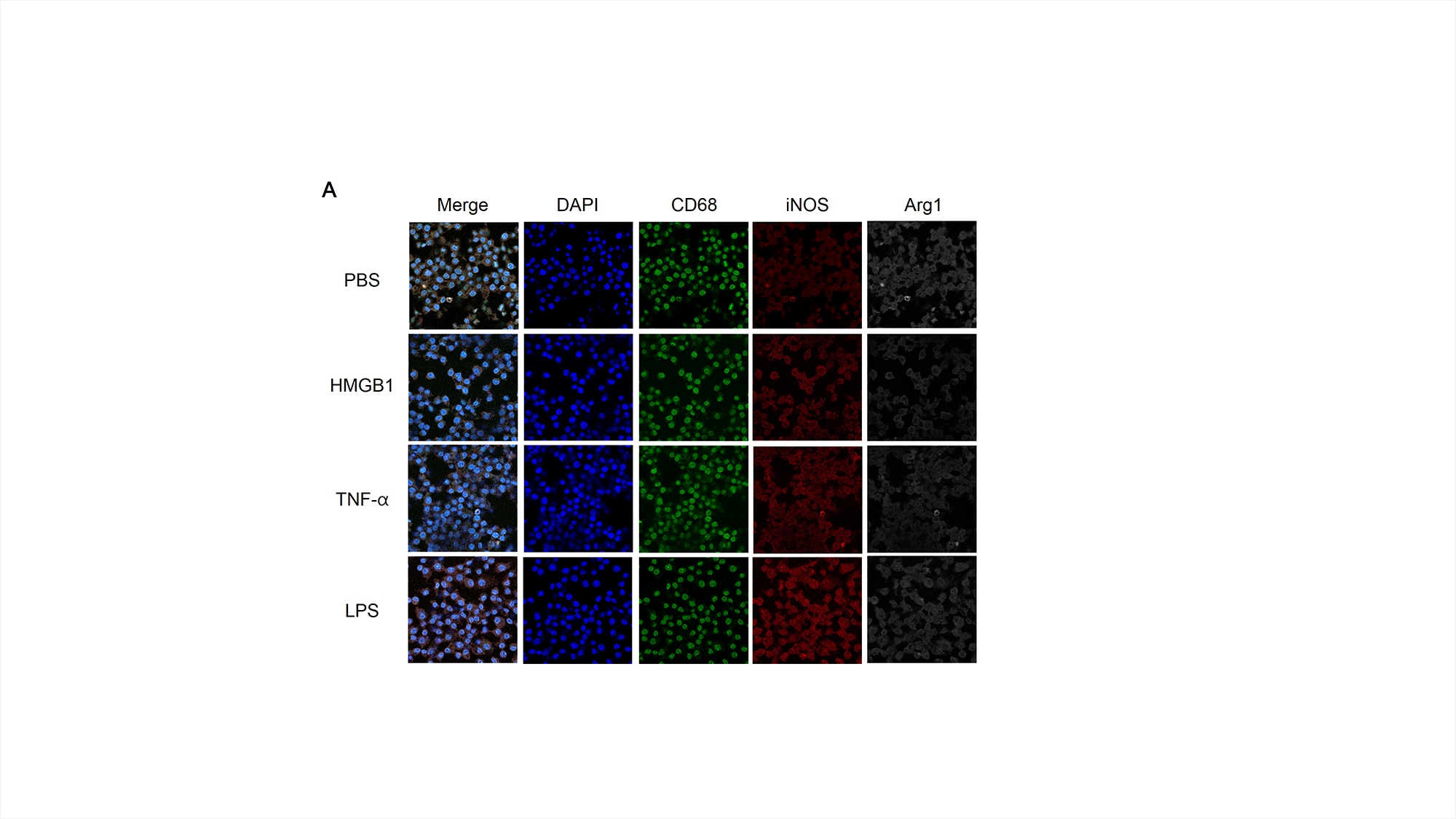

Supplement: S4 Fig — The macrophage content of the RAW264.7 was detected by immunofluorescence (IF) using CD68, inducible nitric oxide synthase (iNOS; M1), and arginase-1 (Arg1; M2) antibodies. A) Representative images of M1/M2 macrophage immunofluorescence in the RAW264.7 of the PBS, HMGB1 (0.5 μg/ml), TNF-α (0.1 μg/ml) and LPS (0.1 μg/ml) groups. Digital images of the cells were scanned using a Zeiss LSM 700. The amplification of 400×. (TIF) [file pone.0193005.s004.tif]

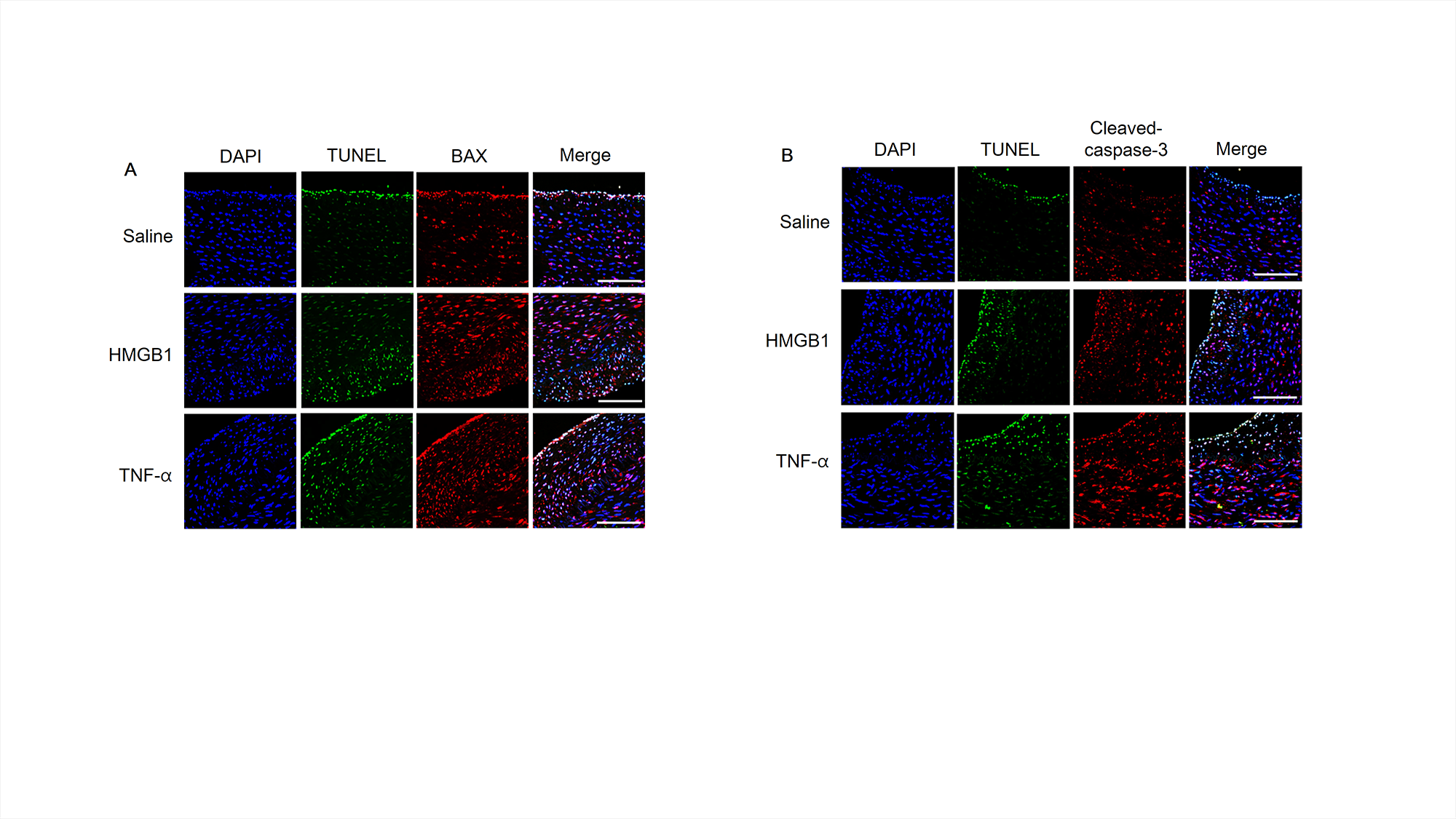

Supplement: S5 Fig — Apoptosis in mini-pig artery plaques from the saline, HMGB1, and TNF-α groups was detected by Determination of apoptosis in plaques using terminal deoxynucleotidyl transferase (TdT)-mediated dUTP nick end labeling (TUNEL) assay and immunofluorescence stain with Bax and cleaved-Caspase-3 antibodies. Representative images of mini-pig arteries from the saline, HMGB1, and TNF-α groups stained with (A) TUNEL and anti-Bax or (B) TUNEL and anti-cleaved-Caspase-3 (amplification 200×). Digital images of the vessels were scanned using a Zeiss LSM 700. Scale bars represent 100 μm. (TIF) [file pone.0193005.s005.tif]
