# Supplementary material for: Macrophage polarization and acceleration of atherosclerotic plaques in a swine model
Source: PLoS One. 2018 Mar 21;13(3):e0193005. doi: 10.1371/journal.pone.0193005 (PMC5862407; doi:10.1371/journal.pone.0193005)
Supplement: S1 Table — I/P, intimal plaque ratio. (PDF) [file pone.0193005.s006.pdf]

S1 Table. Coronary or femoral histology analysis raw data.

Group	Media, mm^2	Intima, mm^2	Plaque, mm^2	I/P, %
**Saline**	1.16	0.51	1.68	30.60
**Saline**	0.78	0.27	1.05	25.92
**Saline**	1.34	0.43	1.77	24.38
**Saline**	1.48	0.37	1.86	20.07
**Saline**	0.56	0.21	0.77	27.21
**Saline**	0.54	0.22	0.76	29.06
**Saline**	1.06	0.33	1.39	23.44
**Saline**	1.01	0.33	1.34	24.41
**Saline**	2.00	0.39	2.40	16.32
**Saline**	1.40	0.25	1.65	15.12
**Saline**	1.06	0.23	1.29	17.70
**HMGB1**	0.51	0.39	0.91	43.23
**HMGB1**	1.12	0.56	1.68	33.18
**HMGB1**	1.33	0.94	2.27	41.21
**HMGB1**	1.35	0.73	2.08	34.94
**HMGB1**	0.81	0.78	1.59	49.02
**HMGB1**	3.53	1.62	5.15	31.44
**HMGB1**	0.77	0.63	1.40	44.91
**HMGB1**	0.87	0.79	1.67	47.61
**HMGB1**	0.65	0.37	1.01	36.18
**HMGB1**	0.65	0.50	1.15	43.07
**HMGB1**	0.54	0.33	0.87	38.30
**TNF-α**	0.61	0.58	1.19	48.55
**TNF-α**	0.86	0.81	1.68	48.43
**TNF-α**	0.98	1.99	2.97	66.96
**TNF-α**	0.62	0.66	1.28	51.29
**TNF-α**	1.13	0.23	1.36	17.04
**TNF-α**	1.42	1.42	2.83	50.02
**TNF-α**	2.50	0.77	3.27	23.66
**TNF-α**	0.82	0.22	1.04	21.54
**TNF-α**	1.23	0.27	1.50	18.04
**TNF-α**	1.93	0.97	2.91	33.48

I/P, intimal plaque ratio
