# Supplementary material for: Macrophage polarization and acceleration of atherosclerotic plaques in a swine model
Source: PLoS One. 2018 Mar 21;13(3):e0193005. doi: 10.1371/journal.pone.0193005 (PMC5862407; doi:10.1371/journal.pone.0193005)
Supplement: S2 Table — RD, reference diameter; MLD, minimal luminal diameter; DS, diameter stenosis. (PDF) [file pone.0193005.s007.pdf]

S2 Table. Coronary or femoral quantitative angiography raw data.

Group	RD, mm	MLD, mm	DS, %
**Saline**	3.16	2.41	23.54
**Saline**	2.73	2.15	21.22
**Saline**	2.34	1.83	21.88
**Saline**	2.1	1.61	23.6
**Saline**	3.02	2.23	26.26
**Saline**	3.52	2.84	19.36
**Saline**	4.39	3.54	19.4
**Saline**	3.41	2.66	21.95
**Saline**	3.55	2.89	18.48
**Saline**	3.34	2.69	19.41
**Saline**	4.00	3.24	18.96
**HMGB1**	2.01	1.32	33.98
**HMGB1**	2.31	1.7	26.53
**HMGB1**	2.47	1.71	30.72
**HMGB1**	2.56	1.54	40.05
**HMGB1**	5.19	3.88	25.21
**HMGB1**	2.86	2.07	27.52
**HMGB1**	3.64	2.57	29.57
**HMGB1**	1.79	1.21	25.16
**HMGB1**	5.89	4.46	23.87
**HMGB1**	2.35	1.51	34.51
**HMGB1**	1.99	1.19	38.54
**TNF-α**	2.56	3	25.51
**TNF-α**	2.13	1.21	43.4
**TNF-α**	2.51	1.47	41.32
**TNF-α**	1.79	1.03	42.6
**TNF-α**	3.85	2.79	27.72
**TNF-α**	4.14	3.16	23.68
**TNF-α**	4.76	3.21	32.57
**TNF-α**	3.66	2.61	28.69
**TNF-α**	4.31	3.27	24.21
**TNF-α**	3.57	2.59	27.42

RD, reference diameter; MLD, minimal luminal diameter; DS, diameter stenosis
